# Validation of commercial ERK antibodies against the ERK orthologue of the scleractinian coral *Stylophora pistillata*

**DOI:** 10.12688/f1000research.11365.2

**Published:** 2017-07-03

**Authors:** Lucile Courtial, Vincent Picco, Gilles Pagès, Christine Ferrier-Pagès

**Affiliations:** 1 Marine Department, Centre Scientifique de Monaco, Monaco, MC-98000, Monaco; 2 Sorbonne Universités, Pierre and Marie Curie University, Paris, 75252, France; 3 Laboratoire d’Excellence, UMR ENTROPIE, Nouméa, 98848, New Caledonia; 4 Biomedical Department, Centre Scientifique de Monaco, Monaco, MC-98000, Monaco; 5 Institute for Research on Cancer and Aging of Nice (IRCAN), University Nice Sophia-Antipolis, CNRS UMR7284/INSERM U1081, Centre Antoine Lacassagne, Nice, 06189, France

**Keywords:** Antibody validation, ERK, Corals, MAPK

## Abstract

The extracellular signal-regulated protein kinase (ERK) signalling pathway controls key cellular processes, such as cell cycle regulation, cell fate determination and the response to external stressors. Although ERK functions are well studied in a variety of living organisms ranging from yeast to mammals, its functions in corals are still poorly known. The present work aims to give practical tools to study the expression level of ERK protein and the activity of the ERK signalling pathway in corals. The antibody characterisation experiment was performed five times and identical results were obtained. The present study validated the immune-reactivity of commercially available antibodies directed against ERK and its phosphorylated/activated forms on protein extracts of the reef-building coral *Stylophora pistillata*.

## Introduction

Mitogen activated protein kinases (MAPKs) are highly conserved proteins involved in signalling pathways and control key cellular processes such as proliferation, differentiation, migration, survival and apoptosis ( Dhillon *et al*., 2007). The MAPK gene family encompasses three major subfamilies: the extracellular signal-regulated kinase (ERK), p38/HOG and c-Jun N-terminal kinase (JNK) groups. The ERK family is the most studied in mammals ( Boulton *et al*., 1990; Dhillon *et al*., 2007) because it is involved in meiosis, mitosis and post mitotic functions in differentiated cells, as well as in the oxidative stress response and wound healing ( Castellano *et al.*, 2014; Johnson & Lapadat, 2002; Matsubayashi *et al*., 2004; Runchel *et al*., 2011). The ERK gene family is evolutionnarily conserved and is found in all eukaryotes, including yeasts, plants, vertebrates and invertebrates ( Chen *et al*., 2001; Widmann *et al*., 1999). Although recent molecular studies have shown the existence of ERK genes in different coral species ( Mayfield *et al*., 2010; Siboni *et al*., 2012; van de Water *et al*., 2015), ERK activity and specific functions are not yet clearly defined. ERK activation occurs through phosphorylation of the Threonine and Tyrosine residues of an ERK-specific TEY motif by the upstream kinases of ERK, the mitogen-activated protein kinase kinase (MAPKK or MEK). ERK phosphorylation on these residues is classically considered the most appropriate readout for the activity of the ERK signalling pathway. However, it has never been monitored in corals. Overall, MAPK activities in corals have only been investigated once, in a study focusing on the JNK subfamily ( Courtial *et al*., 2017).

In this work, we used the scleractinian coral *Stylophora pistillata*, a very abundant species in most tropical reefs ( Veron & Stafford-Smith, 2000). We applied the same protocol as in Courtial *et al*. (2017) to demonstrate the efficiency of antibodies directed against the mammalian phosphorylated forms of ERK (pERK) and total ERK to detect the ERK orthologs in *S. pistillata* ( Table 1). According to the manufacturer’s instructions, the antibody used in this study and directed against the Thr202/Tyr204 di-phosphorylated active ERK (Thermo Scientific Pierce; MA5-15174) showed reactivity with fruit fly, human, mink, mouse, non-human primate, pig, rat and zebrafish. The immunogen used to generate this rabbit IgG monoclonal antibody was a synthetic phosphopeptide corresponding to residues surrounding the phospho-Thr202/Tyr204 of the human p44/ERK1 MAP kinase. This antibody is not cross-reactive with the corresponding phosphorylated residues of either JNK/SAPK or p38. The ERK1/ERK2 antibody (Thermo Scientific Pierce; MA5-15605) used in the study previously showed reactivity with human and mouse samples. The immunogen used to generate this mouse IgG2b monoclonal antibody was a purified recombinant fragment of human MAPK.

**Table 1.** Primary and secondary antibodies.

Antibody	Manufacturer	Catalogue number	RRID	Concentration
p44+42 MAPK (Erk1,2) Antibody (3F8)	Thermo Fisher Scientific	MA5-15605	AB_10983247	1µg/mL (1/1000)
Phospho-p44 MAPK + p42 MAPK pTyr204 Antibody (B.742.5)	Thermo Fisher Scientific	MA5-15174	AB_10980347	1µg/mL (1/1000)
Peroxidase-AffiniPure Goat Anti-Mouse IgG (H+L) antibody	Jackson ImmunoResearch Labs	115-035-003	AB_10015289	1µg/mL (1/10000)
Peroxidase-AffiniPure Goat Anti-Rabbit IgG (H+L) antibody	Jackson ImmunoResearch Labs	111-035-003	AB_2313567	1µg/mL (1/10000)
IRDye 800CW Goat Anti-Rabbit IgG (H+L)	LI-COR Biosciences	926-32211	AB_621843	0.1µg/mL (1/10000)
IRDye 680RD Goat anti-Mouse IgG (H + L)	LI-COR Biosciences	926-68070	AB_10956588	0.1µg/mL (1/10000)

## Methods

### Maintenance of *Stylophora pistillata* nubbins and human fibroblasts

Nubbins of *Stylophora pistillata* were collected from five mother colonies maintained in the aquaria facilities of the Centre Scientifique de Monaco. Two small nubbins (3–5 cm long) were cut off from each mother colony, and were allowed to heal for four weeks in 15 L open system tanks before the experiments. Corals were maintained in the same conditions as the mother colonies, *i.e.* at 25°C, under a photosynthetic active radiation of 200 µmol photon.m ^-2^.s ^-1^ provided by 400 W metal halide lamps (HPIT, Philips) and were fed twice a week with *Artemia salina nauplii*. Seawater in the tanks was continuously renewed at a rate of 10 L.h ^-1^.

Immortalized skin fibroblasts (BJ-EHLT cells) were kindly provided by E. Gilson’s lab (IRCAN) and cultured in Dulbecco’s Modified Eagle’s Medium (Invitrogen, Villebon-sur-Yvette, France) supplemented with 10% heat-inactivated fetal calf serum (Dutscher, Brumath, France) at 37°C in an atmosphere of 5% CO _2_, as previously described ( Biroccio *et al*., 2013).

### UO126 treatment of coral nubbins

Incubations were performed in 100 mL beakers containing one coral nubbin each, and filled with 40 mL of 0.45 μm filtered seawater. They were placed in the dark for one hour in either a control condition containing 0.005% DMSO (vehicle) or a condition with 5 μmol.L ^-1^ UO126 (Selleck Chemicals), a MEK inhibitor ( Tang *et al*., 2003). The incubation medium was continuously stirred using magnetic stirrers at a constant temperature of 25°C. At the end of the incubation, nubbins were frozen and kept at – 80°C prior to western blot analysis.

### UVR and temperature treatment of coral nubbins

Incubations were performed in 100 mL beakers containing one coral nubbin each and filled with 40 mL of 0.45 μm filtered seawater and continuously stirred using magnetic stirrers. High temperature or/and ultraviolet radiation (UVR) stresses (i.e. four environmental conditions: control (at 25°C and without UVR), thermal stress (30°C without UVR), UVR stress (25°C under UVR), thermal and UVR stresses (30°C and under UVR)) were applied to corals and ERK activation was monitored after 30 minutes of stress. Thermal stress corresponded to an increase in temperature from the normal culture condition of 25°C to 30°C. The UVR stress corresponded to an increase in UVR from 0 (HQI lamps in the culture conditions) to a radiation intensity of about 3 W.m ^−2^ UVB and 30 W.m ^−2^ UVA (Q-Panel UVA 340 lamps). At the end of the incubation, nubbins were frozen and kept at – 80°C prior to western blot analysis.

### Western blot analysis

Immuno-detections were performed as in Courtial *et al.* (2017; Table 2 and Table 3). Briefly, nubbins were airbrushed in 1 mL Laemmli buffer (2% SDS, 10% glycerol, 50mM Tris HCL pH7), ( Laemmli, 1970) using an air-pick (5 bars) to remove the totality of the tissues surrounding the skeleton was removed from coral. Samples were then sonicated for 30 seconds, and centrifuged (3 × 5 minutes at 15 000 g) to remove the lipid supernatant and debris. Fibroblasts were washed twice in phosphate buffered saline solution (PBS), lyzed in Laemmli buffer directly in the dishes and sonicated for 30 seconds. The total protein concentration of all samples was determined using a BCA protein Assay Kit (Thermo Fisher Scientific), according to the manufacturer’s recommendation. 1,4 Dithiothreitol (1 mM) and bromophenol blue (0.1%) were added to the samples, which were then heated for 5 minutes at 95°C.

**Table 2.** Tissue extraction and western blot protocol.

Process	Reagent	Manufacturer	Catalogue number	Concentration/Composition
Tissue extraction	Laemmli buffer 1.5X	Homemade		150 mM Tris-HCl pH 7, 25% glycerol, 2% SDS
Sample preparation	Laemmli - 1,4 Dithiothreitol - bromophenol blue solution	Homemade		1.5 X- 50 mM - 0.1%
Electrophoresis	ECL gradient gel 8–16%	GE Healthcare Lifesciences	29-9901-58	
	TG-SDS 10X running buffer	EUROMEDEX	EU0510	1X
Protein transfer	DUNN transfer buffer	Homemade		10 mM NaHCO3 - 3 mM Na2CO3 - 10% Ethanol (pH 9.9)
Blocking	Blocking reagent	Homemade		PBS + milk (3%)
Washes	Wash buffer 10X	Homemade		PBS 10X Tween 20 1N
Membrane coloration	Coloration buffer	Homemade		Isopropanol (25%) + acetic acid (10%) + amido black (0.1%)
Membrane destain	Destain buffer	Homemade		Isopropanol (25%) + acetic acid (10%)
Target detection	Immobilon Western HRP Substrate	Millipore	WBKLS0500	
Reagents	BCA QuantiPro BCA Assay Kit	Sigma-Aldrich	QPBCA-1KT	
	Milk	Itambe ®		
	Methanol	Sigma-Aldrich		

**Table 3.** Reagents for tissue extraction and western blots.

Protocol steps	Reagent	Time	Temperature
Tissue extraction	Laemmli 1.5 X (1mL)		RT
Sonication	Laemmli 1.5 X	30 sec	RT
Centrifugation (x3 15000 g)	Laemmli 1.5 X	3 × 5 min	RT
Addition of 1,4 Dithiothreitol - bromophenol blue solution	Laemmli 1.5 X		RT
Heat up		5 min	95°C
Electrophoresis (100 V)	Running buffer	variable	RT
Transfer (200 mA)	Transfer buffer	overnight	4°C
Coloration	Isopropanol (25%) + acetic acid (10%) + amido black (0.1%)	5 min	RT
Destain	Isopropanol (25%) + acetic acid (10%)	3 × 5 min	RT
Blocking	PBS + milk (3%)	30 min	RT
Primary antibodies	PBS + milk (1%) + ab (1/1000)	overnight	4°C
Washes (3 times)	Wash buffer 1X	3 × 15 min	RT
Secondary antibody	PBS + milk (1%) + ab (1/10000)	2h	RT
Washes (5 times)	Wash buffer 1X	5 × 15 min	RT
Detection	Immobilon Western HRP Substrate	30 sec – 10 min	RT

60 μg of proteins were separated on 10% polyacrylamide gels at 300 mA and 110 V at room temperature. Proteins were then transferred on a PVDF membrane at 4°C overnight in Dunn’s transfer buffer at 200 mA. After a rinse in distilled water, membranes were saturated for 30 minutes in PBS - 3% low fat milk, rinsed in PBS and incubated with primary antibodies diluted in PBS - 1% low fat milk at 4°C overnight. The antibody directed against Thr202/Tyr204 di-phosphorylated active ERK was from Thermo Scientific Pierce (rabbit monoclonal; MA5-15174; batch no. OC1680806); the anti-ERK1/2 antibody was from Thermo Scientific Pierce (mouse monoclonal; MA5-15605; batch no. PH1895491). After extensive washing (4×30 minutes) in PBS – 0.1% Tween 20, membranes were incubated for 2 hours at room temperature in the simultaneous presence of IRDye 680RD goat anti-mouse (925-68070) and IRDye 800CW goat anti-rabbit (925-32211; Li-COR Biotechnology GmbH, Bad Homburg, Germany) secondary antibodies, or with anti-mouse and anti-rabbit HRP-conjugated antibody. Another set of extensive rinsing (4×30 minutes) in PBS – 0.1% Tween 20 was performed before membranes were imaged with an Odyssey device (LI-COR Biosciences, Lincoln, Nebraska) to detect fluorescence and HRP activity using Millipore ECL.

Densitometric analysis of the western blots was performed using Image Studio v2.1 software (Li-COR Biosciences). Intensity of the pERK signal was normalized to the intensity of ERK signal. The relative intensities between control and inhibitor conditions were compared using a t-test. Statistical analysis was done using the software Graphpad Prism v5.03.

## Results and discussion

**Figure 1.**
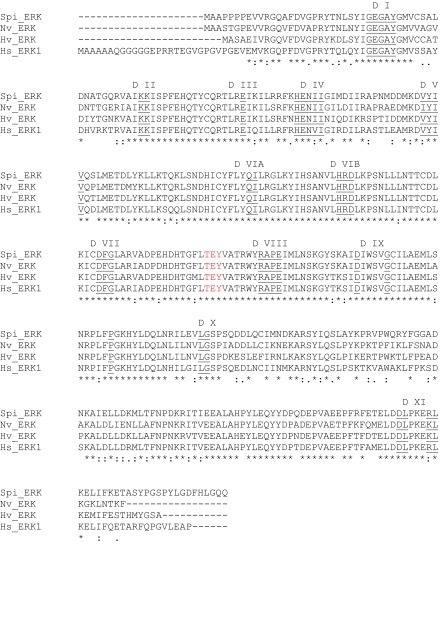
Sequence alignment of MAPK orthologs. The ERK orthologs of *Stylphora pistillata* (Spi-ERK), *Nematostella vectensis* (Nv-ERK), *Hydra vulgaris* (Hv-ERK), and the human ERK1 (Hs-ERK1) protein sequences are shown. The ERK-specific TEY motif is highlighted in red. The eleven conserved kinase domains are underlined.

**Figure 2.**
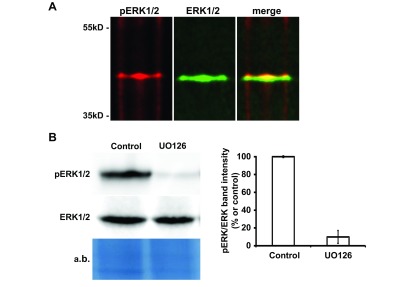
Detection of ERK activity in corals. ( **A**) Fluorescent immunoblot revealing activated (pERK) and total forms of ERK (ERK) present in *Stylphora pistillata* nubbins. Molecular weight standards in kilo Daltons (kDa) are indicated on the left side of the figure. ( **B**) Immunoblot performed with ERK and pERK antibodies on protein extracts from coral nubbins incubated in the absence (Control) or presence of the MEK inhibitor U0126. Densitometric analysis of activated ERK intensities is presented on the right of the figure. The amido black total protein staining of the western blot membrane is shown as a loading control. The medians and standard deviations of three independent experiments are presented (***, p<0.01, t-test).

In order to confirm the presence of an ERK ortholog in corals, the human protein sequence of ERK1 (NP_001035145) was compared to the transcriptome database of *Stylophora pistillata* using the BLAST software ( Altschul *et al.*, 1990; Karako-Lampert *et al*., 2014). An open reading frame was retrieved from the best hit sequence with a predicted molecular weight of 42 kDa ( Spi_isotig05348). This sequence (hereafter referred to as Spi-ERK for *S. pistillata* ERK) is the only one that shows an homology as high as 81%, 80% and 78% with the protein sequences of the cnidarians *Nematostella vectensis* ERK (Nv-ERK; XP_001629498.1), *Hydra vulgaris* ERK (Hv-ERK; XP_002154499.3) and the human MAPK3/ERK1 (Hs-ERK1), respectively ( Figure 1) ( Krishna *et al*., 2013; Putnam *et al*., 2007). These sequences all contain both the conserved kinase domains ( Hanks & Hunter, 1995) and the TEY motif of the catalytic domain, which is unique for ERK orthologs ( Davis, 2000; Figure 1). An interesting point to note is that a unique sequence showing these features is present in *N. vectensis* and *H. vulgaris* genomes, as well as in the *S. pistillata* transcriptome database. This result suggests that a single ortholog of ERK is present in these cnidarians, consistently with previous work where only one ERK ortholog was found ( Castellano *et al.*, 2014; Russo *et al.*, 2004) but as opposed to the two genes encoding ERKs in most mammalian genomes ( Ip & Davis, 1998). Furthermore, based on the high level of sequence conservation between distant species ( Hanks & Hunter, 1995), antibodies directed against portions of the ERK human proteins may recognize ERKs from other species. Accordingly, we detected a single immune-reactive band with the total-ERK antibody by western blot on *S. pistillata* extracts ( Figure 2A and Supplementary Figure S1). Spi-ERK should retain the mechanism of activation by phosphorylation of the Threonine and the Tyrosine residues of the ERK-specific TEY motif. Hence, the MA5-15174 antibody directed against the phosphorylated Thr202 and the Tyr204 ( *i.e.* the phosphorylated TEY motif) should detect a phosphorylated TEY motif of Spi-ERK (phospho-ERK). This is consistent with what we observed, as we detected a unique immune-reactive band of approximately 40 kDa with both antibodies ( Figure 2A).

Interestingly, the fluorescent immunoblot technique showed that the bands detected with the phosphorylated- and the total-ERK antibodies mostly co-migrate, suggesting that the same protein is detected ( Figure 2A). The slight electrophoretic migration shift of the band detected with the anti-phosphorylated ERK antibody would be consistent with the phosphorylation of the threonine and tyrosine residues of the TEY motif as previously described ( Aoki *et al*., 2011). These results suggest that ERK and its phosphorylated form are correctly recognized by the antibodies.

RNAi interference techniques are not available in coral, and the confirmation that the immune reactive bands observed here specifically correspond to ERK could not be obtained through this method. In order to test the specificity of the antibodies, we therefore used U0126, a very potent and selective inhibitor of MEK ( Bain *et al*., 2007). The limited thickness of the animal tissue covering the skeleton and the very large surface of contact of both ectoderm and endoderm with the seawater render *S. pistillata* suitable for treatment with drugs directly diluted in the seawater as we previously showed ( Courtial *et al.*, 2017). U0126 was previously shown to efficiently block MEK activity in a wide variety of organisms, including cnidarians ( Hasse *et al*., 2014; Picco *et al*., 2007; Röttinger *et al.*, 2004). When the inhibitor was added to the seawater, the intensity of the band detected by the anti-total ERK did not vary, while the intensity of the band detected with the anti-phosphorylated ERK antibody was significantly reduced ( Figure 2B and Supplementary Figure S1). Altogether, our results strongly suggest that the proteins detected with the two antibodies were ERK and pERK.

**Figure 3.**
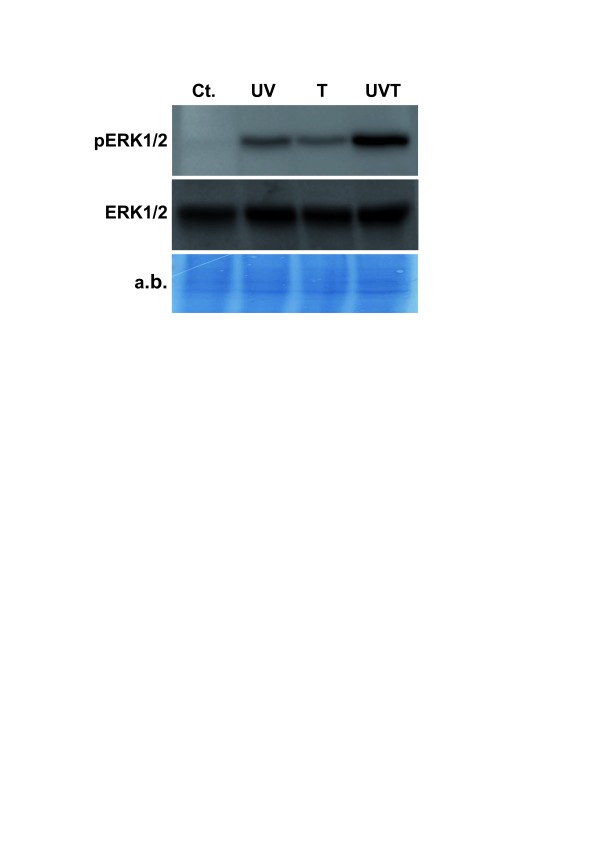
Induction of Spi-ERK phosphorylation by thermal and UV stresses. Immunoblot performed with ERK and pERK antibodies on protein extracts from coral nubbins incubated for 30 minutes in control (Cont.), thermal stress (T), UV stress (UV) or a combination of thermal and UV stresses (UV + T) conditions. The amido black total protein staining of the western blot membrane is shown as a loading control.

To confirm that Spi-ERK activity can dynamically respond to changes in experimental conditions, we performed an induction experiment by modifying culture conditions of the corals. Courtial *et al.* (2017) showed that thermal and UVR stresses induced the formation of reactive oxygen species which are known to trigger ERK phosphorylation ( McCubrey *et al.*, 2006). ERK phosphorylation was enhanced in corals exposed to UVR, high temperature or a combination of both ( Figure 3 and Supplementary Figure S2). These results confirm that the antibodies characterized herein can be used to monitor ERK activity in corals.

**Figure 4.**
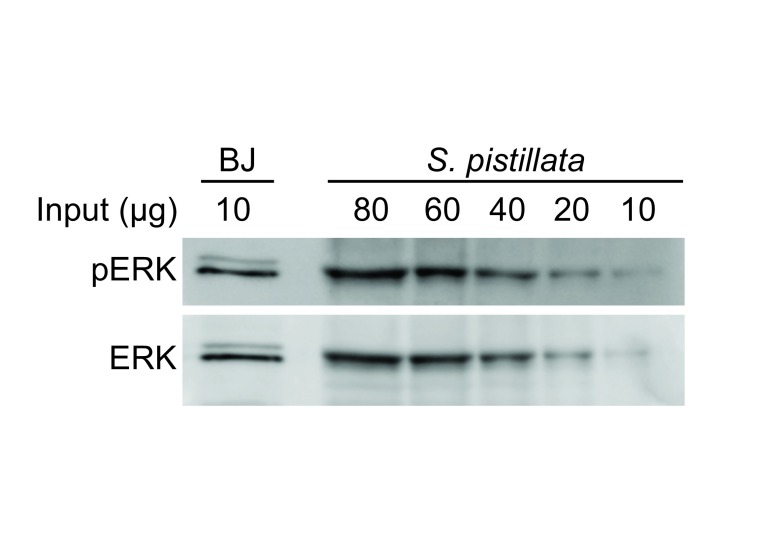
Relative sensitivities of ERK antibodies toward the human and coral proteins. Immunoblot performed with anti-ERK and anti-phospho-ERK on total protein extracts of human fibroblasts (BJ) and *Stylphora pistillata*. The amount of protein loaded in each lane is indicated on the Supplementary Figure S4.

Finally, to assess the performance of these antibodies, we compared the signal obtained on *S. pistillata* and human fibroblasts protein extracts ( Figure 4 and Supplementary Figure S3). We loaded on the same gel 10µg of fibroblast total protein extract and different amounts of *S. pistillata* extracts (ranging from 80 to 10 µg). A signal comparable to the one obtained with the fibroblast extract was observed using 40 µg of coral proteins for both antibodies. This suggests that the affinity of the antibodies towards the coral proteins may be lower than for their human counterparts.

Supplementary Figure S1. Uncropped blot images for Figure 2 and supplementary replicates http://dx.doi.org/10.5256/f1000research.11365.d159188
( **A**) Biological replicates of fluorescent immunoblots performed in control conditions (Ct) are shown (Replicates 1 and 2). The portions of the images used in the main text are outlined. ( **B**) Biological replicates of immunoblots performed on protein extracts from coral nubbins incubated in the absence (Control) or presence of the MEK inhibitor U0126 (UO) (Replicates 1 to 5). The amido black total protein staining of the western blot membrane is shown as a loading control. The portions of the images used in the main text are outlined. Click here for additional data file. Copyright: © 2017 Courtial L et al. 2017 Data associated with the article are available under the terms of the Creative Commons Zero "No rights reserved" data waiver (CC0 1.0 Public domain dedication).

Supplementary Figure S2. Uncropped blot images for Figure 3 and supplementary replicates http://dx.doi.org/10.5256/f1000research.11365.d166821
The portions of the images used in the main text are outlined. Click here for additional data file. Copyright: © 2017 Courtial L et al. 2017 Data associated with the article are available under the terms of the Creative Commons Zero "No rights reserved" data waiver (CC0 1.0 Public domain dedication).

Supplementary Figure S3. Uncropped blot images for Figure 4 and supplementary replicates http://dx.doi.org/10.5256/f1000research.11365.d166825
The portions of the images used in the main text are outlined. Click here for additional data file. Copyright: © 2017 Courtial L et al. 2017 Data associated with the article are available under the terms of the Creative Commons Zero "No rights reserved" data waiver (CC0 1.0 Public domain dedication).

## Conclusion

This work showed that MA5-15174 and MA5-15605 are two specific antibodies that can be used to quantitatively assess *Stylophora pistillata* ERK phosphorylation/activity in different experimental or environmental conditions. We demonstrated the specificity of these antibodies and their good affinity towards their coral targets. It therefore provides the coral research community with a potent tool for the analysis of the activity of a signalling pathway involved in a wide variety of biological processes.

## Data availability

The data referenced by this article are under copyright with the following copyright statement: Copyright: © 2017 Courtial L et al.

Data associated with the article are available under the terms of the Creative Commons Zero "No rights reserved" data waiver (CC0 1.0 Public domain dedication). 

**Supplementary Figure S1. Uncropped blot images for Figure 2 and supplementary replicates.** ( **A**) Biological replicates of fluorescent immunoblots performed in control conditions (Ct) are shown (Replicates 1 and 2). The portions of the images used in the main text are outlined. ( **B**) Biological replicates of immunoblots performed on protein extracts from coral nubbins incubated in the absence (Control) or presence of the MEK inhibitor U0126 (UO) (Replicates 1 to 5). The portions of the images used in the main text are outlined. doi, 10.5256/f1000research.11365.d159188 ( Courtial *et al.*, 2017a).

**Supplementary Figure S2. Uncropped blot images for Figure 3 and supplementary replicates.** The portions of the images used in the main text are outlined. doi, 10.5256/f1000research.11365.d166821 ( Courtial *et al.*, 2017b).

**Supplementary Figure S3. Uncropped blot images for Figure 4 and supplementary replicates.** The portions of the images used in the main text are outlined. doi, 10.5256/f1000research.11365.d166825 ( Courtial *et al.*, 2017c).
