# Supplementary figures and images for: Validation of commercial ERK antibodies against the ERK orthologue of the scleractinian coral Stylophora pistillata
Source: F1000Res. 2017 Jul 3;6:577. Originally published 2017 Apr 26. [Version 2] doi: 10.12688/f1000research.11365.2 (PMC5482343; doi:10.12688/f1000research.11365.2)

**A**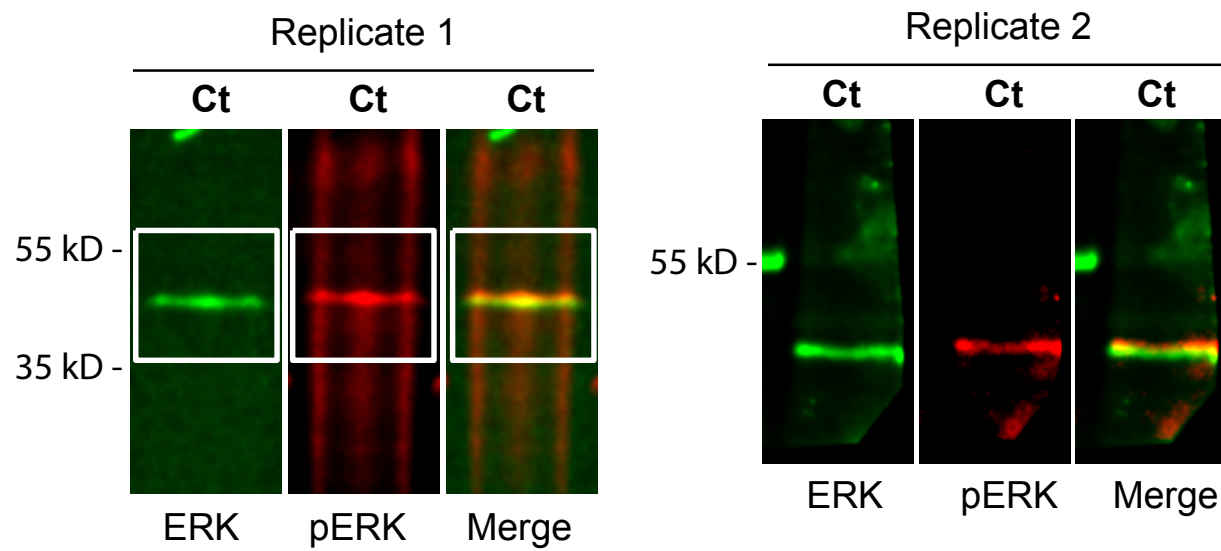**B**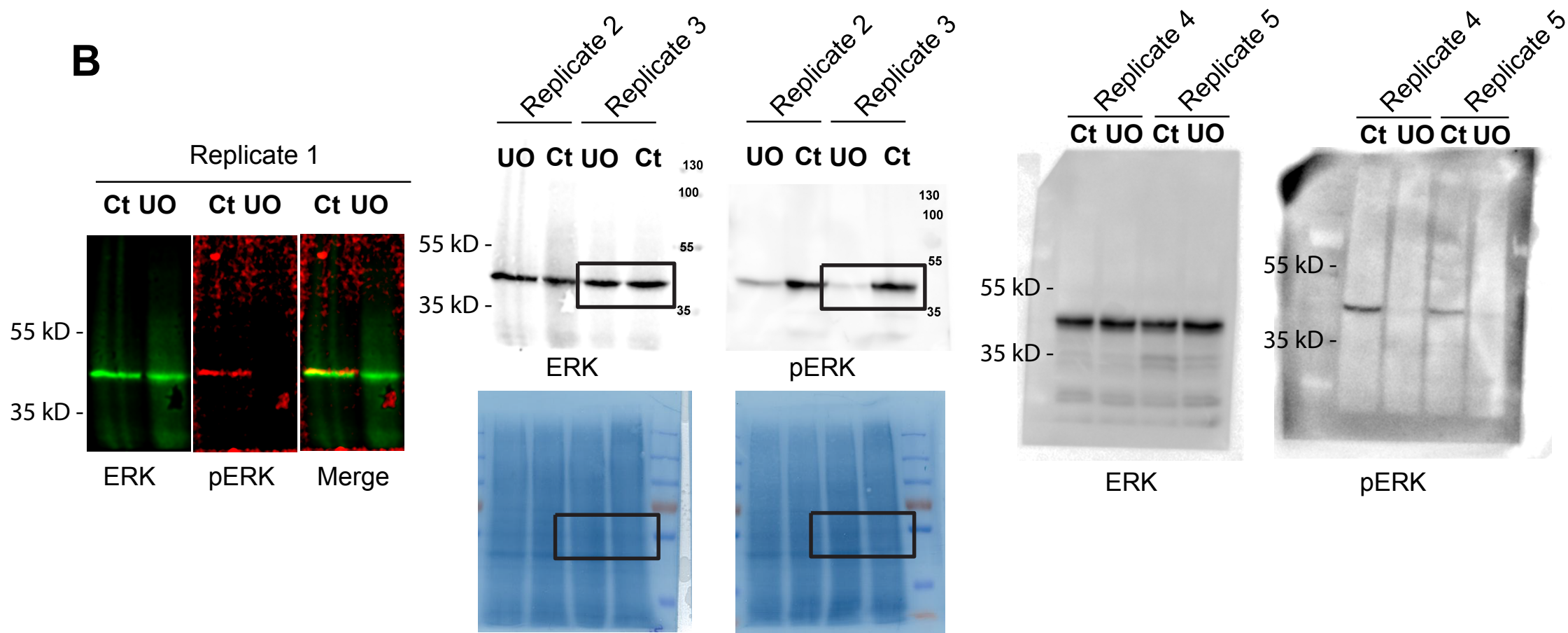

Supplement: Supplementary Figure S1. Uncropped blot images for Figure 2 and supplementary replicates — http://dx.doi.org/10.5256/f1000research.11365.d159188 ( A) Biological replicates of fluorescent immunoblots performed in control conditions (Ct) are shown (Replicates 1 and 2). The portions of the images used in the main text are outlined. ( B) Biological replicates of immunoblots performed on protein extracts from coral nubbins incubated in the absence (Control) or presence of the MEK inhibitor U0126 (UO) (Replicates 1 to 5). The amido black total protein staining of the western blot membrane is shown as a loading control. The portions of the images used in the main text are outlined. [file f1000research-6-13040-s0000.tgz › da516ae1-be3f-46c8-9193-f36306f0ef94_Supplementary_Figure_S1.pdf]

## Replicate 1

Ct UV T UV+T

55 kD -

35 kD -

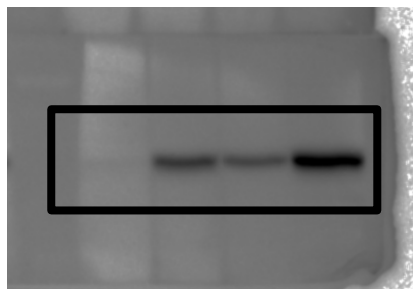

pERK

55 kD -

35 kD -

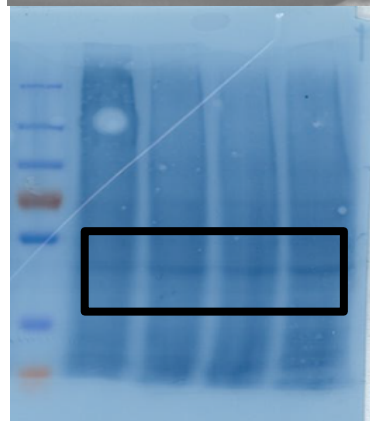

## Replicate 2

Ct UV T UV+T

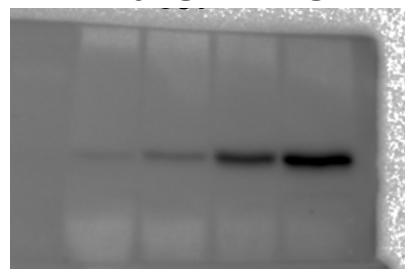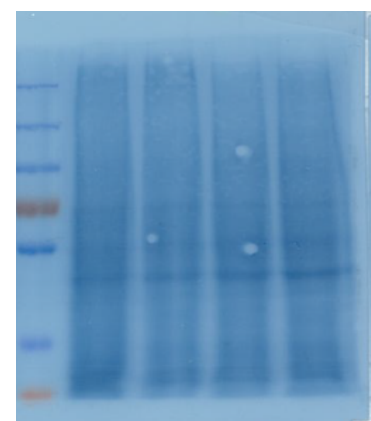

## Replicate 3

Ct UV T UV+T

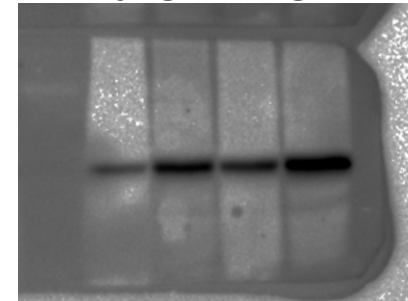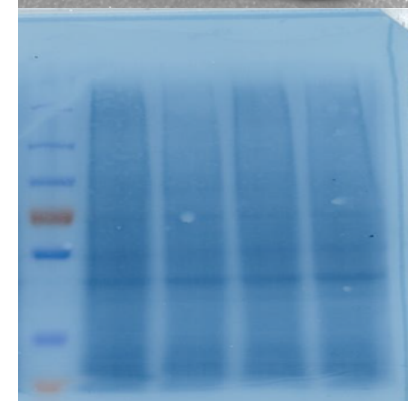

ERK

55 kD -

35 kD -

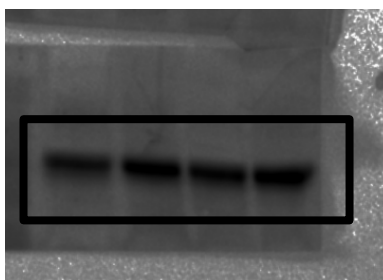

55 kD -

35 kD -

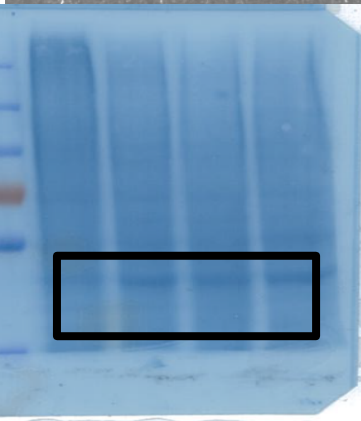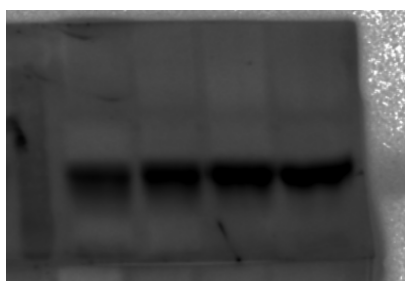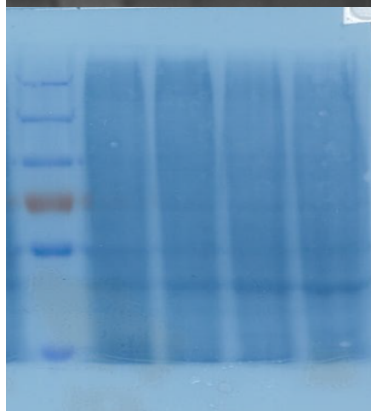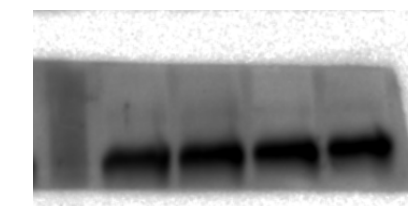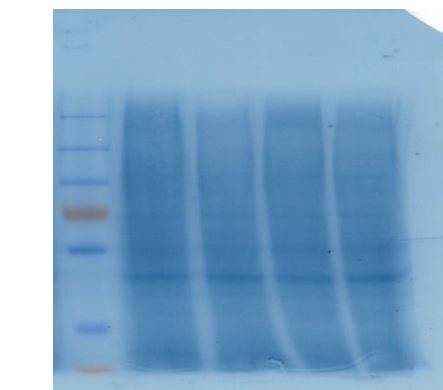

Supplement: Supplementary Figure S2. Uncropped blot images for Figure 3 and supplementary replicates — http://dx.doi.org/10.5256/f1000research.11365.d166821 The portions of the images used in the main text are outlined. [file f1000research-6-13040-s0001.tgz › 60177294-b987-47e2-bfe8-8365b5ac13e2_Supplementary_Figure_S2.pdf]

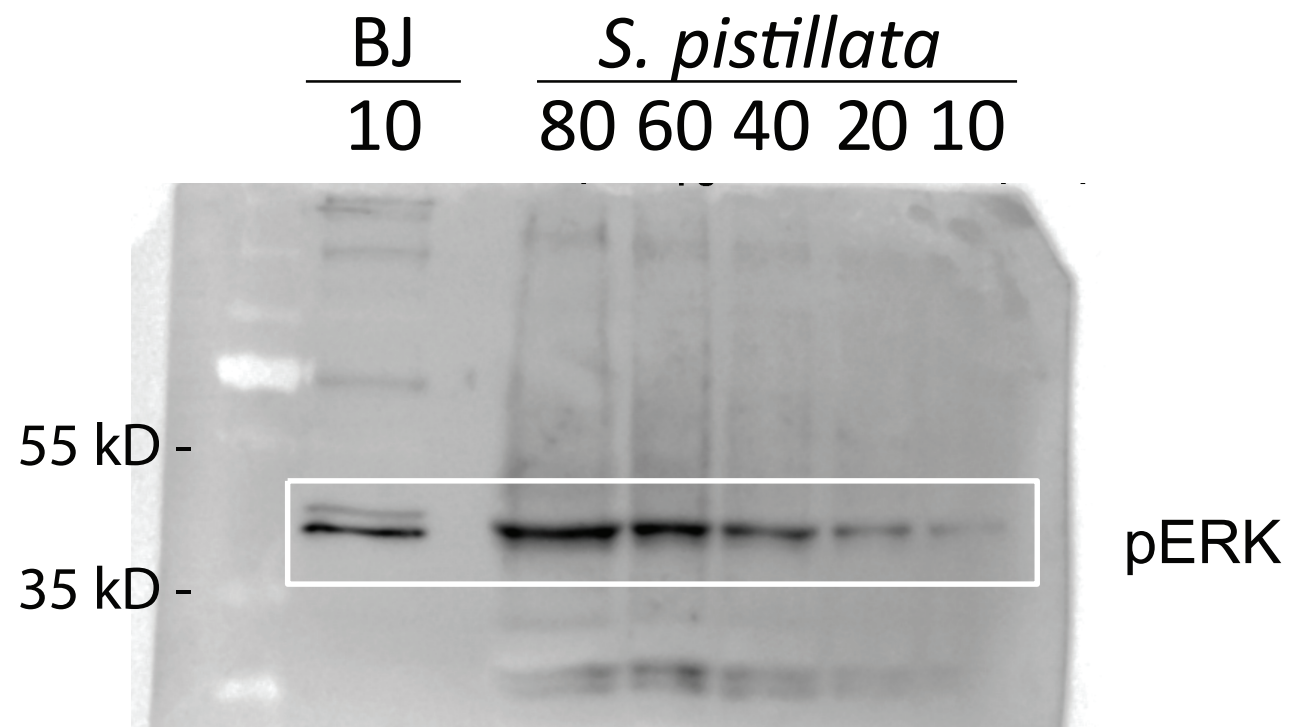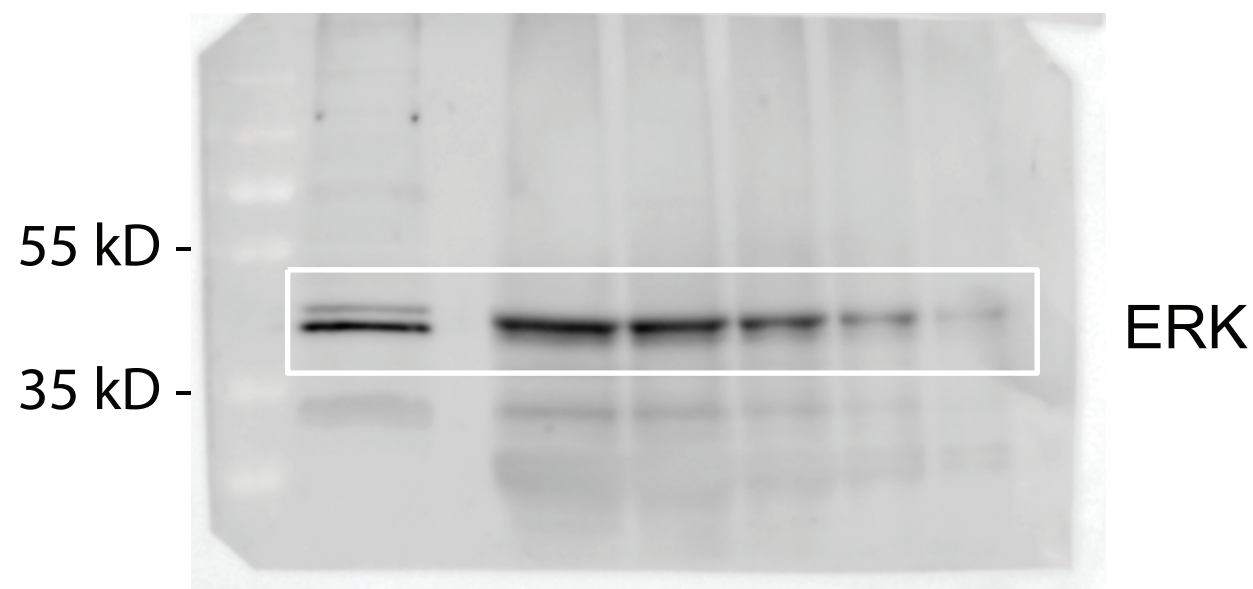

Supplement: Supplementary Figure S3. Uncropped blot images for Figure 4 and supplementary replicates — http://dx.doi.org/10.5256/f1000research.11365.d166825 The portions of the images used in the main text are outlined. [file f1000research-6-13040-s0002.tgz › b69ea011-3679-4ecd-80e4-57344b35932a_Supplementary_Figure_S3.pdf]
