# Supplementary material for: Validation of commercial ERK antibodies against the ERK orthologue of the scleractinian coral Stylophora pistillata
Source: F1000Res. 2017 Jul 3;6:577. Originally published 2017 Apr 26. [Version 2] doi: 10.12688/f1000research.11365.2 (PMC5482343; doi:10.12688/f1000research.11365.2)
Supplement: Peer review history

# Peer review: Referee response for version 2

**DOI:** 10.5256/f1000research.13040.r23990

**Published:** 2017-07-21

**Authors:** María L. Parages

**Affiliations:** 1 Department of Ecology, Faculty of Sciences, University of Málaga, Málaga, Spain

After author response, I recommend the manuscript for indexing. So I suggest the new status as Approved.

I have read this submission. I believe that I have an appropriate level of expertise to confirm that it is of an acceptable scientific standard.

# Peer review: Referee response for version 2

**DOI:** 10.5256/f1000research.13040.r24020

**Published:** 2017-07-04

**Authors:** Andrea Pitzschke

**Affiliations:** 1 Department of Cell Biology and Physiology, University of Salzburg, Salzburg, Austria

In the revised version Courtial et al. have addressed my concerns and considered my suggestions in sufficient detail. I therefore recommend the manuscript for indexing.

I have read this submission. I believe that I have an appropriate level of expertise to confirm that it is of an acceptable scientific standard.

# Peer review: Referee response for version 1

**DOI:** 10.5256/f1000research.12269.r23149

**Published:** 2017-06-22

**Authors:** María L. Parages

**Affiliations:** 1 Department of Ecology, Faculty of Sciences, University of Málaga, Málaga, Spain

In this manuscript the authors Courtial et al. validate the use of commercial ERK antibodies for the detection of MAPK-like proteins in Coral. Although the paper is well written and has the quality to be indexed, a few issues should be figure out before its final acceptance.

 In terms of samples preparation and to allow future replication by others researchers would be appropriate to give more information about how to prepare the samples. For example, “Briefly, coral tissue was removed from the skeleton in 1 mL Laemmli buffer” so, how much coral tissue will be dissolved and resupended it in 1 mL of Laemmli Buffer? Less than 0.5 gr? More?

 While it is true that the authors note that immune-detection were performed as in Courtial *et al.* (2017), and that DTT (Dithiothreitol) and BFB (Bromophenol blue) were added to the samples and heated (5 minutes at 95°) before loading the gels, it is not specified how tissue extraction was performed. I must assume that the Lysis Buffer used was Laemmli Buffer? And in this case, how they have been unable to detect phosphorylated ERK? As far as I know lysis buffer for phosphorylated proteins usually contents EDTA or EGTA to chelate Mg2+/Ca2+,  DTT for reduction of disulfide bonds, serine protease inhibitor (Aprotinin/Leupeptine), phosphatase inhibitors to block dephosphorylation like Na orthovanadate, or Beta-glycerophosphate (false substrate for phosphatase, between others… They also keep everything ice cold? Also, it surprises me that they used 3% low fat milk for membranes blocking, due to also screws up phosphor-tyrosine detection.

 Regarding the presence of an ERK ortholog in coral, the authors show the sequence with code Spi_isotig05348 (Spi_ERK) has the best hit with the human ERK protein (NP_0011035145) and they make reference to Liew *et al.,* 2014. I was looking for this sequence into this paper, and I could not find it. The authors should provide in which database is the transcriptome, as well as, the Spi_ERK sequence itself.

I have read this submission. I believe that I have an appropriate level of expertise to confirm that it is of an acceptable scientific standard, however I have significant reservations, as outlined above.

# Response

**Published:** 2017-06-28

**Authors:** Lucile Courtial

**Affiliations:** Centre Scientifique de Monaco, Monaco

***In this manuscript the authors Courtial et al. validate the use of commercial ERK antibodies for the detection of MAPK-like proteins in Coral. Although the paper is well written and has the quality to be indexed, a few issues should be figure out before its final acceptance.***

 We thank Dr. Parages for her comments and suggestions. To answer her concerns, we have added information and replaced a citation in the manuscript as detailed bellow. 

***In terms of samples preparation and to allow future replication by others researchers would be appropriate to give more information about how to prepare the samples. For example, “Briefly, coral tissue was removed from the skeleton in 1 mL Laemmli buffer” so, how much coral tissue will be dissolved and resupended it in 1 mL of Laemmli Buffer? Less than 0.5 gr? More?***

 All of the tissue from 3-5cm long *S. pistillata* nubbins was used. We added the precision in the materials and methods:

 P4: “Two small nubbins (3-5 cm long) were cut off from each mother colony and were allowed to heal for four weeks in 15 L open system tanks before the experiments.”

 P5: “Briefly, nubbins were airbrushed in 1 mL Laemmli buffer (2% SDS, 10% glycerol, 50mM Tris HCL pH7) ( Laemmli 1970) using an air-pick (5 bars) to remove the totality of the tissues surrounding the skeleton was removed from coral.”

**While it is true that the authors note that immune-detection were performed as in Courtial *et al.* (2017), and that DTT (Dithiothreitol) and BFB (Bromophenol blue) were added to the samples and heated (5 minutes at 95°) before loading the gels, it is not specified how tissue extraction was performed. I must assume that the Lysis Buffer used was Laemmli Buffer?**

**And in this case, how they have been unable to detect phosphorylated ERK? As far as I know lysis buffer for phosphorylated proteins usually contents EDTA or EGTA to chelate Mg2+/Ca2+,  DTT for reduction of disulfide bonds, serine protease inhibitor (Aprotinin/Leupeptine), phosphatase inhibitors to block dephosphorylation like Na orthovanadate, or Beta-glycerophosphate (false substrate for phosphatase, between others…**

 Coral tissues were indeed lyzed in laemmli buffer. To prevent any doubt when reading the methods, we added a precision in the text:

 P3: “Briefly, nubbins were airbrushed in 1 mL Laemmli buffer (2% SDS, 10% glycerol, 50mM Tris HCL pH7) (Laemmli 1970) using an air-pick (5 bars) to remove the totality of the tissues surrounding the skeleton.”

 To answer the concern of Dr. Parages about the possible phosphatase activity in the samples, we would like to specify the fact that, due to a high concentration of SDS, the Laemmli buffer is a strongly denaturing buffer for proteins, including phosphatases. Therefore, this buffer, commonly used at room temperature, preserves phosphorylation of the proteins without the need to add phosphatases inhibitors (see Picco et al. 2016 for example).  

***They also keep everything ice cold? Also, it surprises me that they used 3% low fat milk for membranes blocking, due to also screws up phosphor-tyrosine detection.***

 We fully agree with Dr. Parages, the use of milk as a blocking buffer is usually not recommended for the detection of phospho-proteins as it may contain phospho-proteins that can interact with the anti-phospho primary antibodies. However, we have successfully used this blocking buffer in diverse experimental setups, including human cultured cells, ascidian embryos as well as coral lysates. We used this buffer in the course of this study because it gives far less background noise than any other blocking buffers tested.

***Regarding the presence of an ERK ortholog in coral, the authors show the sequence with code Spi_isotig05348 (Spi_ERK) has the best hit with the human ERK protein (NP_0011035145) and they make reference to Liew et al., 2014. I was looking for this sequence into this paper, and I could not find it. The authors should provide in which database is the transcriptome, as well as, the Spi_ERK sequence itself.***

 We are grateful to the reviewer for questioning this particular point as it allowed us to uncover a significant error in the citation we used.

 The reference for the Spi EST containing the ERK orthologue open reading frame was obtained from the database generated during the study by Karako-Lampert, Zoccola et al. (Plos One 2014) and not the one by Liew et al (p.7) . The database containing the sequence can be downloaded from this address: http://data.centrescientifique.mc/CSMdata-stylodata.html. As mentioned in the manuscript, the ERK1 human protein sequence was blasted (tblastn) against the Karako-Lampert database using the blast tool hosted on a publically accessible local server of the Centre Scientifique de Monaco ( http://data.centrescientifique.mc/blast/blast.php). The correct reference has been added to the manuscript.

 Due to current major security concerns for our servers, we have chosen not to include the aforementioned URL in the present manuscript. However, this URL is openly disclosed in the Karako-Lampert et al. paper, which should allow readers to access the database without the needing to contact corresponding authors.

# Peer review: Referee response for version 1

**DOI:** 10.5256/f1000research.12269.r23532

**Published:** 2017-06-16

**Authors:** Immacolata Castellano

**Affiliations:** 1 Department of Biology and Evolution of Marine Organisms, Stazione Zoologica Anton Dohrn, Naples, Italy

The paper by Courtial et al. describes the cross-reactivity of two commercial antibodies produced against the mammalian forms of ERK for the scleractinian coral Stylophora pistillata. This should open new perspective for the study of ERK signalling in response to different  environmental cues.

 The paper is clear and well written, however it lacks of some references.

 In the Introduction, the authors should cite other invertebrates where ERK signalling is known to be conserved and regulated by environmental cues, for example Ciona intestinalis (Castellano et al, PlOS One 2014, Castellano et al, Open Biology 2015). Similarly, in Results and Discussion, when the authors say that .. "a single orthologue of ERK is present in these cnidarians, as opposed to the two genes encoding ERKs in most mammalian genomes”, they should specify that also in other invertebrates, only one ERK form was found (Russo et al, JBC 2004; Castellano et al, PloS One 2014). Also the use of the MEK inhibitor U0126 was assessed in C. intestinalis (Castellano et al, Open Biology 2015).

 Finally the authors pay attention along the text to some errors, i.e. change “Thr2020/204” with Thr202/204, and “through this medium” with “through this method”.

I have read this submission. I believe that I have an appropriate level of expertise to confirm that it is of an acceptable scientific standard.

# Response

**Published:** 2017-06-28

**Authors:** Lucile Courtial

**Affiliations:** Centre Scientifique de Monaco, Monaco

***The paper by Courtial et al. describes the cross-reactivity of two commercial antibodies produced against the mammalian forms of ERK for the scleractinian coral Stylophora pistillata. This should open new perspective for the study of ERK signalling in response to different environmental cues.***

******

***The paper is clear and well written, however it lacks of some references.***

 We thank Dr. Castellano for her comments and suggestions. We have added information to the manuscript to answer her concerns as detailed bellow.

***In the Introduction, the authors should cite other invertebrates where ERK signalling is known to be conserved and regulated by environmental cues, for example Ciona intestinalis (Castellano et al, PlOS One 2014, Castellano et al, Open Biology 2015).***

 We added a precision and the reference in the text: “The ERK gene family is evolutionnarily conserved and is found in all eukaryotes, including yeasts, plants, vertebrates and invertebrates (Widmann et al. 1999; Chen et al. 2001; Castellano et al. 2014).”

***Similarly, in Results and Discussion, when the authors say that .. "a single orthologue of ERK is present in these cnidarians, as opposed to the two genes encoding ERKs in most mammalian genomes”, they should specify that also in other invertebrates, only one ERK form was found (Russo et al, JBC 2004; Castellano et al, PloS One 2014).***

 We added the precision in the text:P4 “This result suggests that a single ortholog of ERK is present in these cnidarians, consistently with previous work where only one ERK ortholog was found (Russo et al. 2004; Castellano et al. 2014)  but as opposed to the two genes encoding ERKs in most mammalian genomes (Ip and Davis 1998).”

***Also the use of the MEK inhibitor U0126 was assessed in C. intestinalis (Castellano et al, Open Biology 2015).***

 Despite extensive search into the reference cited by Dr. Castellano, we could not find any experiment using U0126 in this paper. The aforementioned reference instead reports the use of a dual specificity phosphatase inhibitor. Moreover, the work of Picco et al. (2007) cited in the manuscript already reports the use of U0126 in *Ciona* embryos. We therefore did not include the suggested citation in the text.

***Finally the authors pay attention along the text to some errors, i.e. change “Thr2020/204” with Thr202/204, and “through this medium” with “through this method”.***

 We changed the errors in the revised manuscript.

# Peer review: Referee response for version 1

**DOI:** 10.5256/f1000research.12269.r22707

**Published:** 2017-05-30

**Authors:** Andrea Pitzschke

**Affiliations:** 1 Department of Cell Biology and Physiology, University of Salzburg, Salzburg, Austria

The manuscript provides first insight into a putative MAPK in the coral Stylophora pistillata.

 Experiments include protein extraction and immunoblot analysis.

 Overall: The experiments that had been performed are properly designed. However, the manuscript lacks sufficiently-detailed information, as well as controls (protein loading). Conclusions are premature or should be re-phrased.

 Detailed points of criticism:

 Title “orthologue” is inappropriate. Should be “homologue”.

**Methods**

 P3 “small rubbins selected” – please be more specific about size and sampling:

 “tissue removed from coral”: be more specific. Tissue primarily from the surface, how deep was the cut into the material? (I suggest to include a schematic figure incl. scale-bar). This information is important because inhibitors (e.g. UO126) will only diffuse over a short distance, i.e. not reach deeper layers.

 P4: “extensive washing”: duration and number of solution changes missing

 Fig.2B: “% or control” rather OF control. The error bar in the control sample is irrelevant, as it is defined as strictly 100%.

 There is no documentation of protein loading (e.g. Coomassie-stained membrane after immunodetection). The U126-independent intensity of the ERK-Signal is insufficient as control.

 Conclusions: “…antibody can be used…in different experimental or environmental conditions”

 This conclusion is premature. As a minimum, the authors should perform an induction experiment. The inhibitory approach (U126) only evidences that a MAPKK is the upstream regulator. Coral research community will only benefit from the antibody and the current study if dynamic ERK activity responses can be monitored.

I have read this submission. I believe that I have an appropriate level of expertise to confirm that it is of an acceptable scientific standard, however I have significant reservations, as outlined above.

# Response

**Published:** 2017-06-28

**Authors:** Lucile Courtial

**Affiliations:** Centre Scientifique de Monaco, Monaco

***Overall: The experiments that had been performed are properly designed. However, the manuscript lacks sufficiently-detailed information, as well as controls (protein loading). Conclusions are premature or should be re-phrased.***

 We thank Dr. Pitzschke for her comments and suggestions. We have added information to the manuscript and performed an additional experiment to answer her concerns as detailed bellow.

***Detailed points of criticism:***

******

***Title “orthologue” is inappropriate. Should be “homologue”.***

 We have used the term “orthologue” in our manuscript in its definition notably given by Walter Fitch (Fitch 1970, 2000), that is: “Orthology is that relationship where sequence divergence follows speciation, that is, where the common ancestor of the two genes lies in the common ancestor of the taxa from which the two sequences were obtained”.

 Dr. Pitzschke is right, the Spi- and Hs-ERK proteins are homologues but, on top of that, they also are orthologues. We therefore still think that the term “orthologue” is more accurate in our case.

***Methods***

***P3 “small nubbins selected” –please be more specific about size and sampling:***

***“tissue removed from coral”: be more specific. Tissue primarily from the surface, how deep was the cut into the material?***

 To be more specific, we added the following sentences in the revised manuscript:

 P2: “Two small nubbins (3-5 cm long) were cut off from each mother colony and were allowed to heal for four weeks in 15 L open system tanks before the experiments.”

 P3: “Briefly, nubbins were airbrushed in 1 mL Laemmli buffer ( *i.e.* lysing buffer, 1.5 X, Laemmli 1970) using an air-pick (5 bars) to remove the totality of the tissues surrounding the skeleton was removed from coral.”

***This information is important because inhibitors (e.g. UO126) will only diffuse over a short distance, i.e. not reach deeper layers.***

 We added a sentence to prevent further doubts concerning the bioavailability of U0126.

 P6: “In order to test the specificity of the antibodies, we therefore used U0126, a very potent and selective inhibitor of MEK (Bain et al. 2007). The limited thickness of the animal tissue covering the skeleton and the very large surface of contact of both ectoderm and endoderm with the seawater render S. pistillata suitable for treatment with drugs directly diluted in the seawater as we previously showed (Courtial et al. 2017) …”

***P4: “extensive washing”: duration and number of solution changes missing***

 We added precisions in the materials and methods:  P4 : “4 x 30 minutes”

***Fig.2B: “% or control” rather OF control. The error bar in the control sample is irrelevant, as it is defined as strictly 100%.***

***There is no documentation of protein loading (e.g. Coomassie-stained membrane after immunodetection). The U126-independent intensity of the ERK-Signal is insufficient as control.***

 We added the amido black colored membranes in Figure 2 and Figure S1 as a loading control. 

***Conclusions: “…antibody can be used…in different experimental or environmental conditions”***

***This conclusion is premature. As a minimum, the authors should perform an induction experiment. The inhibitory approach (U126) only evidences that a MAPKK is the upstream regulator. Coral research community will only benefit from the antibody and the current study if dynamic ERK activity responses can be monitored.***

 We performed an additional experiment and added a figure and related text in the manuscript to justify our statement (P9).
